# Limiting Injury During Saphenous Vein Graft Preparation For Coronary Arterial Bypass Prevents Metabolic Decompensation

**DOI:** 10.1038/s41598-017-13819-w

**Published:** 2017-10-27

**Authors:** Joyce Cheung-Flynn, Jun Song, Igor Voskresensky, Eric S. Wise, Yapu Liu, Yanhua Xiong, Susan S. Eagle, Colleen M. Brophy, C. Robb Flynn

**Affiliations:** 1 Department of Surgery, Vanderbilt University Medical Center, Nashville, Tennessee United States of America; 2 People’s Hospital of Puyang, Puyang, China; 3 Department of Anesthesiology, Vanderbilt University Medical Center, Nashville, Tennessee United States of America

## Abstract

Standard harvest and preparation of human saphenous vein (HSV) for autologous coronary and peripheral arterial bypass procedures is associated with injury and increased oxidative stress that negatively affect graft performance. In this study we investigated the global metabolomic profiles of HSV before (unprepared; UP) and after standard vein graft preparation (AP). AP-HSV showed impaired vasomotor function that was associated with increased oxidative stress, phospholipid hydrolysis and energy depletion that are characteristic of mechanical and chemical injury. A porcine model (PSV) was utilized to validate these metabolomic changes in HSV and to determine the efficacy of an improved preparation technique (OP) using pressure-regulated distension, a non-toxic vein marker, and graft storage in buffered PlasmaLyte solution in limiting metabolic decompensation due to graft preparation. Deficits in vasomotor function and metabolic signature observed in AP-PSV could be largely mitigated with the OP procedure. These findings suggest that simple strategies aimed at reducing injury during graft harvest and preparation represents a straightforward and viable strategy to preserve conduit function and possibly improve graft patency.

## Introduction

Arterial bypass grafting is performed to improve blood flow to the heart (coronary artery bypass grafting; CABG) and extremities (peripheral arterial bypass grafting; PBG). The human saphenous vein (HSV) is the most commonly used bypass conduit for autotransplantation. After surgical harvest, the HSV is prepared to prevent links and kinking upon for implantation. Common, standard vein graft preparation (AP) for these patients includes distention using a hand held syringe to identify leaks. The intraluminal pressure imparted by manual distension often exceeds 300 mmHg and causes physiologic impairment of the graft^1^ and pathologic responses of vascular tissue *in vivo* ^2^ and *in vitro* ^1,3^. Once distended, “roadmap” inking of HSV with off-label use of standard surgical markers is often used to orient the graft prior intraoperative storage in non-buffered, acidic saline solution until implantation. The gentian violet and isopropyl alcohol common to surgical skin markers are cytotoxic and have been shown to reduce smooth muscle contractility and endothelial-dependent relaxation^4,5^.

Graft failure rates have been reported to be as high as 45% in CABG and 39% in PBG in large prospective multi-institutional PREVENT trials^6,7^. While insufficient adaptation or remodeling of the vein to the arterial circulation contributes to graft failure, it is long recognized that technical elements play a role in determining clinical outcomes of the vein graft^8–11^. Intimal hyperplasia, a ‘response to injury’ is the leading cause of graft failure^12^. While incompletely understood, intimal hyperplasia results from a cascade of molecular and cellular events that are triggered by injury. This complex process involving inflammation, vascular smooth muscle proliferation, migration, phenotypic modulation, and extracellular matrix production leads to intimal thickening and in some instances lumen occlusion that contributes to vein graft failure^13,14^.

The AP technique contributes to reduced viability, impaired vasomotor function, increased oxidative stress, and *in vitro* intimal thickening of the HSV^1,4,15–17^, suggesting that vein graft preparation injury prior to implantation plays in role in eliciting the ‘response to injury’. An improved preparation technique (OP) using pressure-regulated distension, a non-toxic vein marker, and storage in buffered PlasmaLyte solution has been shown to decrease injury in a porcine saphenous vein (PSV) model^17,18^. PSV prepared with the OP technique maintain normal vasomotor function and viscoelastic properties suggesting that changes in vein graft preparation could reduce injury to the HSV^17,18^.

Metabolic changes in tissues and cells levels are the most proximal reporters of physiologic status and precede downstream transcriptional, posttranscriptional, translational, and posttranslational events. Advances in analytical technologies and bioinformatics capabilities have enabled the identification and measurement of thousands of metabolites simultaneously using targeted or untargeted “metabolomics” analyses. Such analyses reflect the true functional endpoints of biological events and may plausibly establish correlations between vein graft injury and metabolic changes. There is great utility in defining the early metabolic changes in response to vascular injury. Such findings may guide strategies to prevent injury during vein graft preparation.

The hypothesis of this investigation was that HSV injury during vein graft preparation leads to acute metabolic changes, preceding physiological decompensation that contributes to vascular injury response. We further hypothesized that preventing this injury would preserve vein graft function. Therefore, we implemented untargeted, discovery-based metabolomics analyses to identify differences between the metabolic profiles of HSV conduits that were collected before (UP) and after standard preparation (AP) from the same patients. To validate these findings we utilized the porcine saphenous vein (PSV) model of vein graft preparation which allows for standardization of graft preparation approaches^17^. Porcine is a well-established large animal model for addressing vascular graft biology in that porcine saphenous PSV has similar caliber to HSV^19–21^. We characterized functional responses and compared metabolomic signatures of UP- and AP-PSV to those generated after the optimized preparation (OP).

## Materials and Methods

All chemicals were purchased from Sigma-Aldrich unless otherwise specified.

### Procurement of HSV

Subjects gave informed written consent before participating in this study, which was approved by the Internal Review Board of Vanderbilt University (090607). All studies were conducted in accordance with NIH and institutional guidelines for human subject research. The study protocol conformed to the ethical guidelines of the 1975 Declaration of Helsinki, as reflected in a priori approval by Vanderbilt University Medical School.

Human saphenous vein (HSV) (n = 15) was obtained from patients undergoing CABG procedures. From each patient, segments were collected immediately after surgical harvest (UP) and after standard intraoperative graft preparation according to the surgeon’s discretion and standard of care, before implantation (AP). Veins were collected in heparinized (10 U/mL) Plasma-Lyte (Baxter Healthcare) solution (HP; one liter has an ionic concentration of 140 mEq sodium, 5 mEq potassium, 3 mEq magnesium, 98 mEq chloride, 27 mEq acetate, and 23 mEq gluconate) and transported to the laboratory within 10 min. The HSV was tested immediately for physiologic function in the muscle bath, formalin fixed for immunohistochemistry, and snap-frozen in liquid nitrogen for metabolomic analyses. The average time between UP and AP segment collection was 3 1 ± 1.1 hr.

### Collection of Clinical Demographic Variables

Demographic variables were collected, including age, sex, race, body mass index, medical comorbidities, pre-operative laboratory values, and preoperative medication regimen.

### Model of vein graft preparation using PSV

Animal procedures followed study protocols approved by the Vanderbilt Institutional Animal Care and Use Committee and adhered to National Institutes of Health guidelines for care and use of laboratory animals. Yorkshire/Landrace pigs weighing 40–45 kg (Oak Hill Genetics, Ewing, IL) were anesthetized and PSV were harvested from the lateral aspect of the lower extremity. The subcutaneous fat and fascia were carefully dissected to expose the PSV with care taken to minimize trauma to the vein. Branches were ligated away from the vein. The PSVs were then explanted and unprepared tissue (UP) was cut from these segments. PSV were then prepared by standard (AP) or the improved (OP) graft preparation as described previously^17^. Briefly, AP-PSV were distended with 10 mL heparinized, normal saline solution (HS, Baxter) using a 60-ml syringe and marked in a continuous line using a standard surgical skin marker (Richard-Allan). The vein was then stored in HS for 1 hr. OP-PSV were distended with an in-line pressure release valve, which limits distension pressure to 140 mmHg^1^, with HP. After distension, the vein was marked in a continuous line using the marker containing FCF (Vasoprep, NJ) and stored in HP for 1 hr at room temperature.

### Physiologic Measurements of Vasocontractility and Vasorelaxation

Force measurements were obtained using Radnoti transducer (model 159901 A) interfaced with PowerLab data acquisition system and chart software (AD Instruments) as described previously^22^. All chemicals were purchased from Sigma-Aldrich unless otherwise specified. Briefly, 1 to 2-mm rings were cut from segments of saphenous veins and suspended in a muscle bath containing bicarbonate buffer (120 mM sodium chloride, 4.7 mM potassium, 1.0 mM magnesium sulfate, 1.0 mM monosodium phosphate, 10 mM glucose, 1.5 mM calcium chloride, 25 mM sodium bicarbonate, pH 7.4) equilibrated with 95% oxygen and 5% carbon dioxide at 37 °C for 2 hours. Rings were contracted first with 110 mM potassium chloride to determine smooth muscle functional viability. Viable tissues were further evaluated for contraction to the contractile agonist phenylephrine (PE; 5 × 10^−6^ M, a dose that produce sub-maximal contraction, 60–80% of the maximal 110 mM potassium-induced contraction). PE pre-contracted tissues were then treated with 5 × 10^−7^ M carbachol or sodium nitroprusside (10^−7^ M) to determine maximal endothelial-dependent and endothelial-independent relaxation responses, respectively. Contractile responses were defined by stress, calculated using force generated: Stress (×10^5^ N/m^2^) = Force (g) × 0.0987/area, where area = wet weight (mg)/ at maximal length (mm)]/1.055. Relaxation responses were calculated as a percentage of the maximal PE contraction. Each data point was averaged from at least two rings from the same specimen.

### Untargeted, global metabolomics

Full details of the methodology for the mass spectrometry-based metabolomics analyses are given in see Supplementary Methods or as described previously^23–25^. Briefly, frozen tissues were subjected to methanol extraction, split into aliquots for analysis by ultrahigh performance liquid chromatography mass spectrometry (UPLC-MS) and gas chromatography mass spectrometry (GC-MS). Internal standards and controls for signal blank, technical replicates, and instrument performance were spiked into the samples and tracked throughout the analysis. Metabolite concentrations were determined by automated ion detection, manual visual curation, and were analyzed in-line using software developed by Metabolon^26^.

### Immunomorphometric analysis of PSV

Additional PSV (n = 4) were obtained and prepared with the AP and OP techniques as described for the PSV model above. Tissues were then formalin-fixed, paraffin-embedded, and stained using Movat’s stain to visualize histomorphometry. Additional tissue sections were stained using the avidin-biotinylated peroxidase complex (ABC) method (Vector lab, Burlingame, CA). Antigen retrieval of sections was performed with citrate buffer (pH 6) at 95 °C for 5 min. After preincubation with 5% goat serum to block nonspecific sites, sections were incubated with primary antibodies against biotinylated lectin (Dolichos biflorus agglutinin; Vector lab) and eNOS (Abcam, Cambridge, MA) to examine endothelial integrity, and 4-HNE (Abcam), MDA (Abcam), and nitrotyrosine (Millipore, Bedford MA) to evaluate oxidative stress-induced protein adduct formation. Biotinylated IgG (Vector lab) was used as secondary antibody at 10 ng/ml. Sections stained for eNOS and lectin were counterstained with hematoxylin (Vector Lab) for 5–15 sec. Tissue sections from the same animal were stained on the same day for the same antigens to control for technical variations. Whole slide imaging was performed in the Digital Histology Shared Resource at Vanderbilt University Medical Center (www.mc.vanderbilt.edu/dhsr).

### Statistics and Bioinformatics

Data were analyzed using GraphPad Prism 7.01 (La Jolla, CA) n and presented as box-and-whisker plots. Paired Student’s *t*-tests were used for binary comparisons. One-way ANOVA test with Tukey post-test was used for three-way comparisons. A *p*-value of <0.05 was considered significant.

Hierarchical clustering, optimizing sample order by Pearson correlation using the average linkage clustering option, was performed using MultiExperiment Viewer (version 4.8.0; http://www.tm4.org). Unsupervised principal component analysis (PCA) was used for the overview of individual and all classes jointly, to observe clustering or separation trends and for the identification of outliers. PCA loading plots were used to identify metabolites responsible for any separation observed. Supervised multivariate analysis was carried out with Orthogonal Partial Least Squares Discriminant Analysis (OPLS-DA) using the MultiBase 2015 (Numerical Dynamics; Tokyo Japan) plugin for Microsoft Excel (Redmond, WA).

## Results

### Patient clinical demographics

Fifteen patients were included in this study. The clinical demographic variables for the patients are typical for patients undergoing CABG procedures (see Supplementary Table S1).

### Standard vein graft preparation impairs vasocontractility in HSV

**Figure 1:**
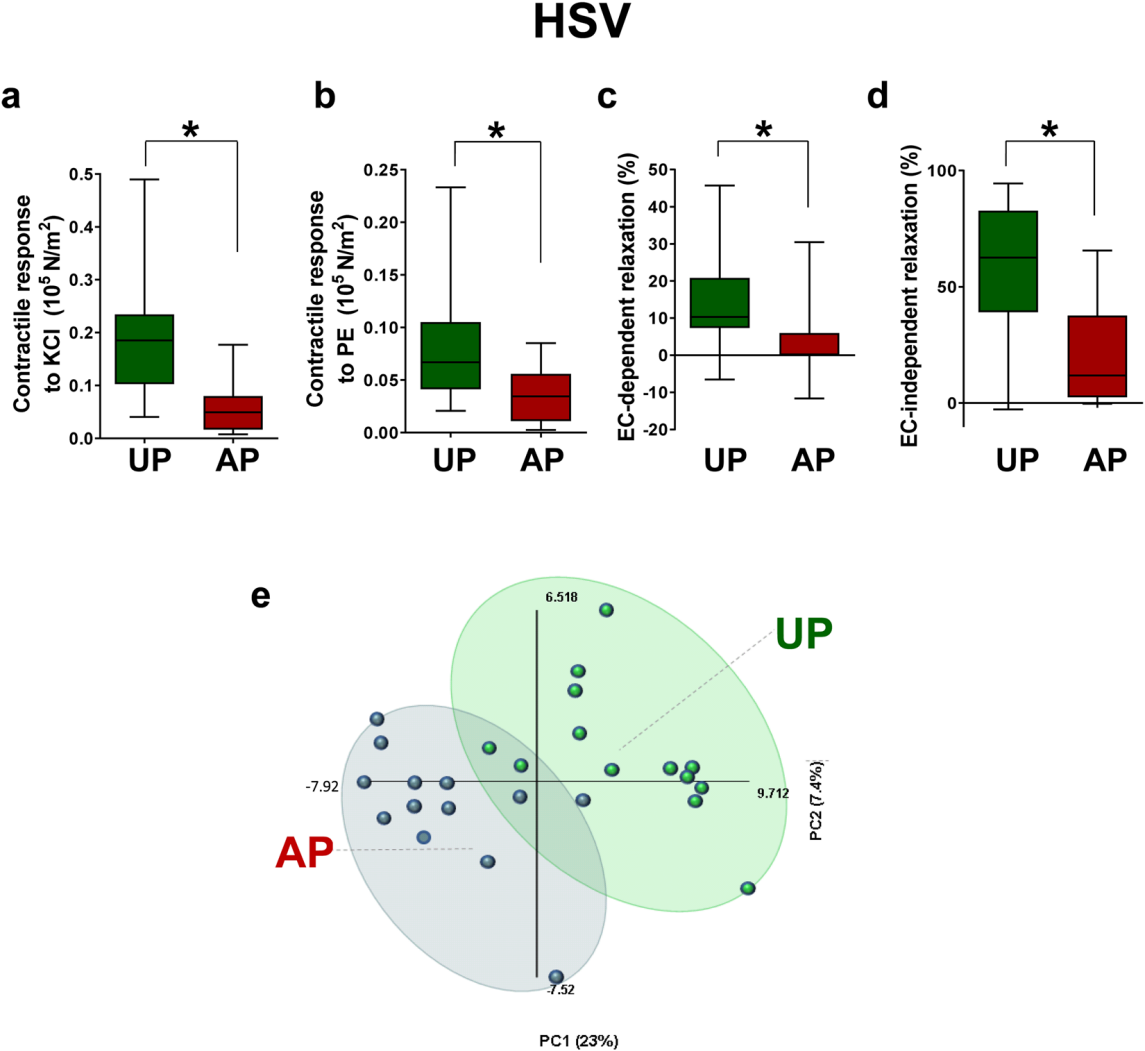
Vasomotor function and targeted metabolomic profiles in unprepared (UP) or after standard preparation (AP) human saphenous vein (HSV). Paired HSV segments (n = 15) were collected from CABG patients and vasomotor function determined in a muscle bath. Contractile responses to (**a**) 110 mM KCl and (**b**) 5 × 10^−6^ M PE in UP- and AP-HSV. (**c**) Endothelial cell- (EC−) dependent relaxation (%) and (**d**) EC-independent relaxation (%) in PE-precontracted UP- and AP-HSV. (**e**) Principle components plots and (**f**) hierarchical clustering of tissue metabolite profiles (n = 13) from UP- and AP-HSV. Values are expressed as box-and-whisker plots, *p ≤ 0.05.

Standard vein graft preparation (AP) reduced contractility to depolarizing KCl, or functional viability of HSV (Fig. 1a). Vasocontractile response to PE were also reduced in AP compared to UP tissues (Fig. 1b). Additionally, endothelial-dependent and –independent responses to CCH and SNP, respectively, were also reduced in AP-HSV confirming that standard graft preparation leads to impaired vasomotor responses (Fig. 1c and d).

### Global metabolic alteration in HSV after standard vein graft preparation

**Figure 2:**
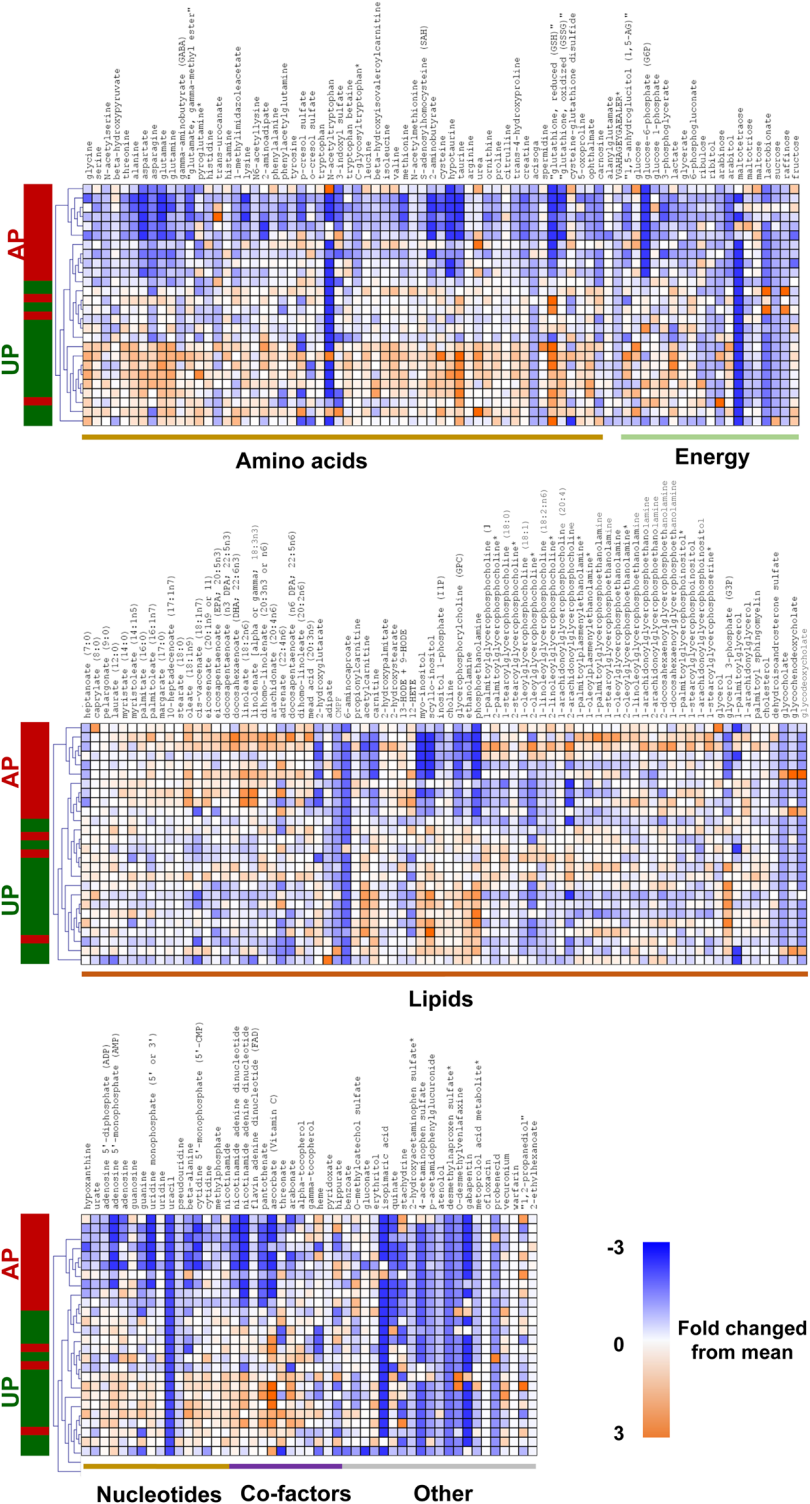
The metabolome of human saphenous vein. Hierarchical clustering analysis of human saphenous vein metabolites identified in unprepared (UP) vein or veins after standard preparation (AP). Heatmap colors in shades of blue indicate an increase relative to the metabolite mean, while orange shades indicated a decrease relative to the metabolite mean. Values shaded in grey were not detected. N = 13 per group.

To identify novel biomarkers that associated with vein graft injury, the metabolic changes of UP and AP HSV were characterized. Untargeted, global metabolomics profiling of HSV permitted comparisons of the abundance of 218 small molecule metabolites that could be confidently identified. Preliminary unsupervised principal component analysis (PCA) revealed two sample pairs in PCA scores plot outside the 0.95 Hotelling’s T2 confidence interval. In both pairs, the UP sample and in one pair the AP sample had several (>30) metabolite abundance values wherein the observed value exceeded the standard deviation >3 fold. These signals were identified as artifacts of sample preparation and prompted us to exclude these two sample pairs from the study (see Supplement data for additional information on these metabolites). After removal of these two sample pairs, the PCA scores plot for the metabolomics subset data showed a reasonable clustering of UP-HSV samples that was distinct from AP-HSV samples (Fig. 1e). Unsupervised hierarchical clustering (Fig. 2) revealed AP-HSV tissues with reductions in the level of cellular antioxidants (6 metabolites), accompanied by increased phospholipid hydrolysis and lipid peroxidation (79 metabolites) and a decrease in amino acids and central energy metabolism (98 metabolites) (Table 1). Of the 216 metabolites detected, 98 showed significant changes (p ≤ 0.05) in AP-HSV compared to UP-HSV.

**Table 1:** Biochemical pathways and metabolite alterations in human saphenous vein after preparation for CABG.

Biochemical pathways	Metabolite Alterations in AP tissues
Cellular antioxidants	↓Glutathione (GSH, GSSG), 5-oxoproline
	↑GSSG/GSH
	↓taurine, urate
Phospholipid hydrolysis and peroxidation	↑Free fatty acids
	↑Lysophospholipids, e.g. arachidonoyl- and linoleyl-glycerophospholipid
	↑Lipid peroxidation products, e.g. 12-HETE, 4-HNE
Central energy metabolism	↓Glycolytic intermediates, e.g. glucose-6-phosphates
	↓Adenylate nucleotides
	↓TCA cycle intermediates, e.g. succinate
	↓co-factors NADH, FAD, NAD

### The AP preparation technique caused vasomotor impairment and metabolic derangement in the PSV model

**Figure 3:**
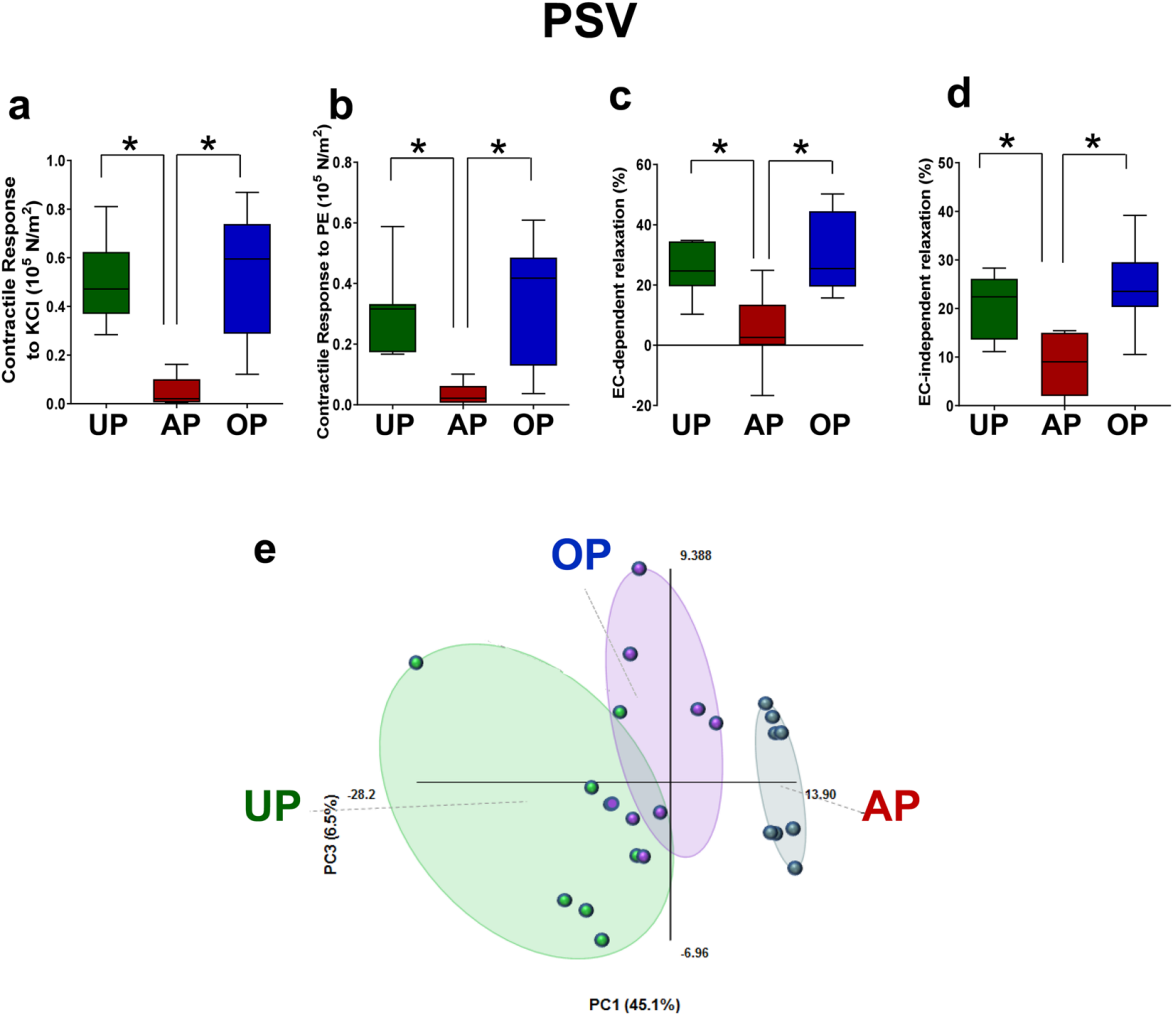
Vasoresponsiveness and targeted metabolomic profiles of unprepared (UP), after standard preparation (AP) or after optimized preparation (OP) in the porcine vein graft preparation model. Freshly isolated porcine saphenous vein (PSV; n = 8) were prepared using the PSV graft preparation model and vasomotor function determined in a muscle bath. Contractile responses to (**a**) 110 mM KCl and (**b**) 5 × 10^−6^ M PE in PSV. (**c**) Endothelial cell- (EC−) dependent relaxation (%) and (**d**) EC-independent relaxation (%) in PE-precontracted PSV. (**e**) Principle components plots and (**f**) hierarchical clustering of tissue metabolite profiles (n = 8) from UP-, AP-, and OP-PSV. Values are expressed as box-and-whisker plots. *p ≤ 0.05.

**Figure 4:**
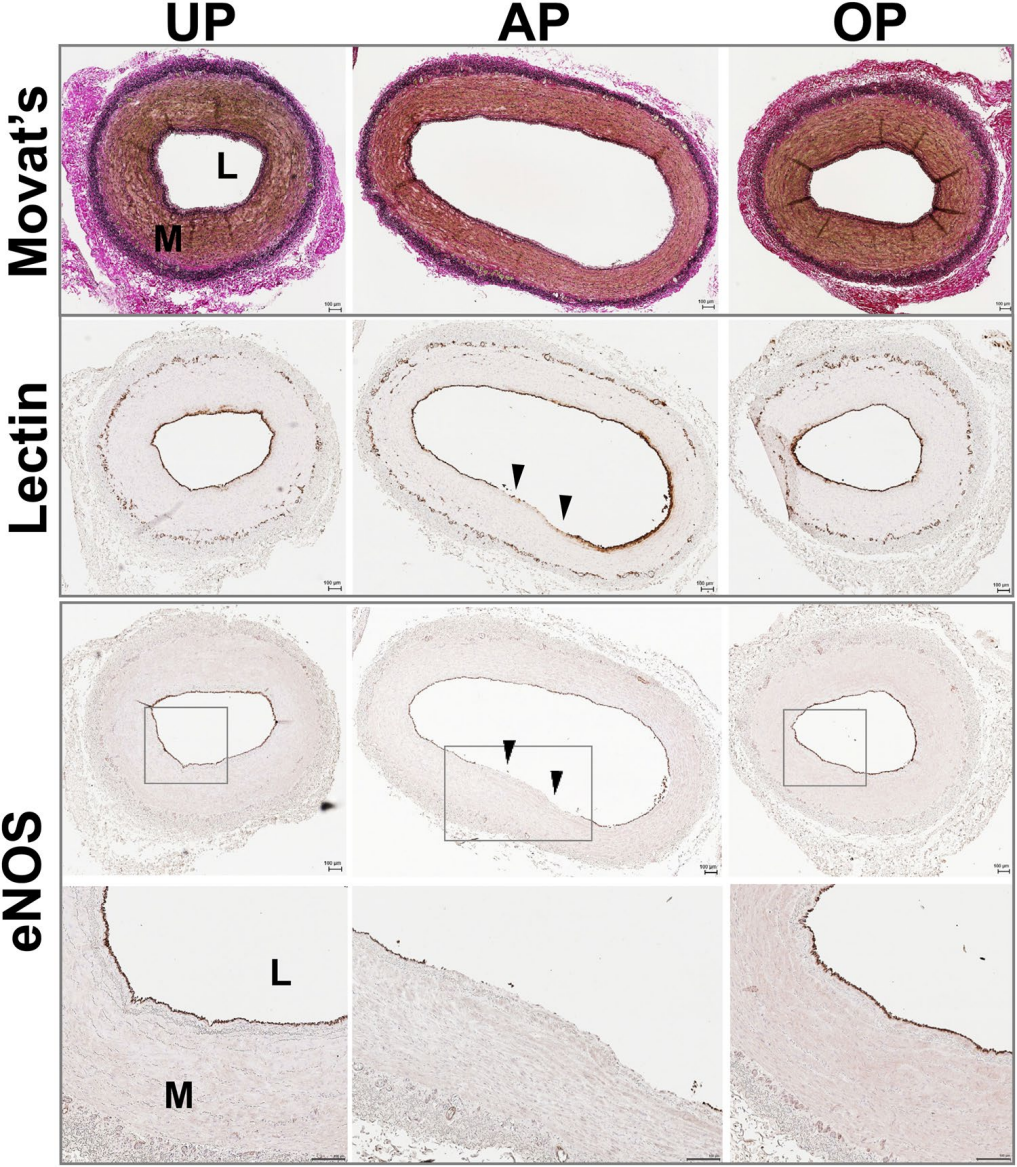
Immunohistomorphology of the porcine saphenous vein before and after vein graft preparation in the PSV model. PSV (n = 4) were harvested and prepared using the AP and OP technqiues, formalin-fixed and paraffin-embedded. Tissue sections were stained with Van Geison’s stain to visualize histomorphology (top row), or lectin (second row) and antibody against endothelial nitric oxide synthase (eNOS, third and fourth row) to examine endothelial integrity. Whole slide imaging was performed at 20X. Fourth row show enlarged area indicated for eNOS staining. Scale bar = 100 $\mu$m. Brown = positive staining. Representative images of staining from 1 pig shown. UP, unprepared; AP, after standard vein graft preparation technique; OP, after optimized vein graft preparation technique, L, lumen; M, medial layer. Arrows indicate endothelial disruption observed in the AP tissues.

To validate the metabolic response of the AP vein graft preparation routine of HSV, a PSV vein graft preparation model^17^ was employed and global metabolite profiles of the PSV (n = 8) prepared using standard (AP) or an improved (OP) vein graft preparation techniques were characterized. AP-PSV exhibited loss in vasomotor function similar to that observed in AP-HSV (Fig. 3a–d). Histological staining (Van Gieson’s or Movat’s stain) of reveals luminal distortion in the AP-PSV (Fig. 4 and Supplementary Figs S3–S5), but not in the UP-PSV. Additionally, endothelial disruption was evident in the AP-PSV as indicated by lectin and eNOS staining (Fig. 4). No difference was detected in level of eNOS except where endothelium was denuded.

**Figure 5:**
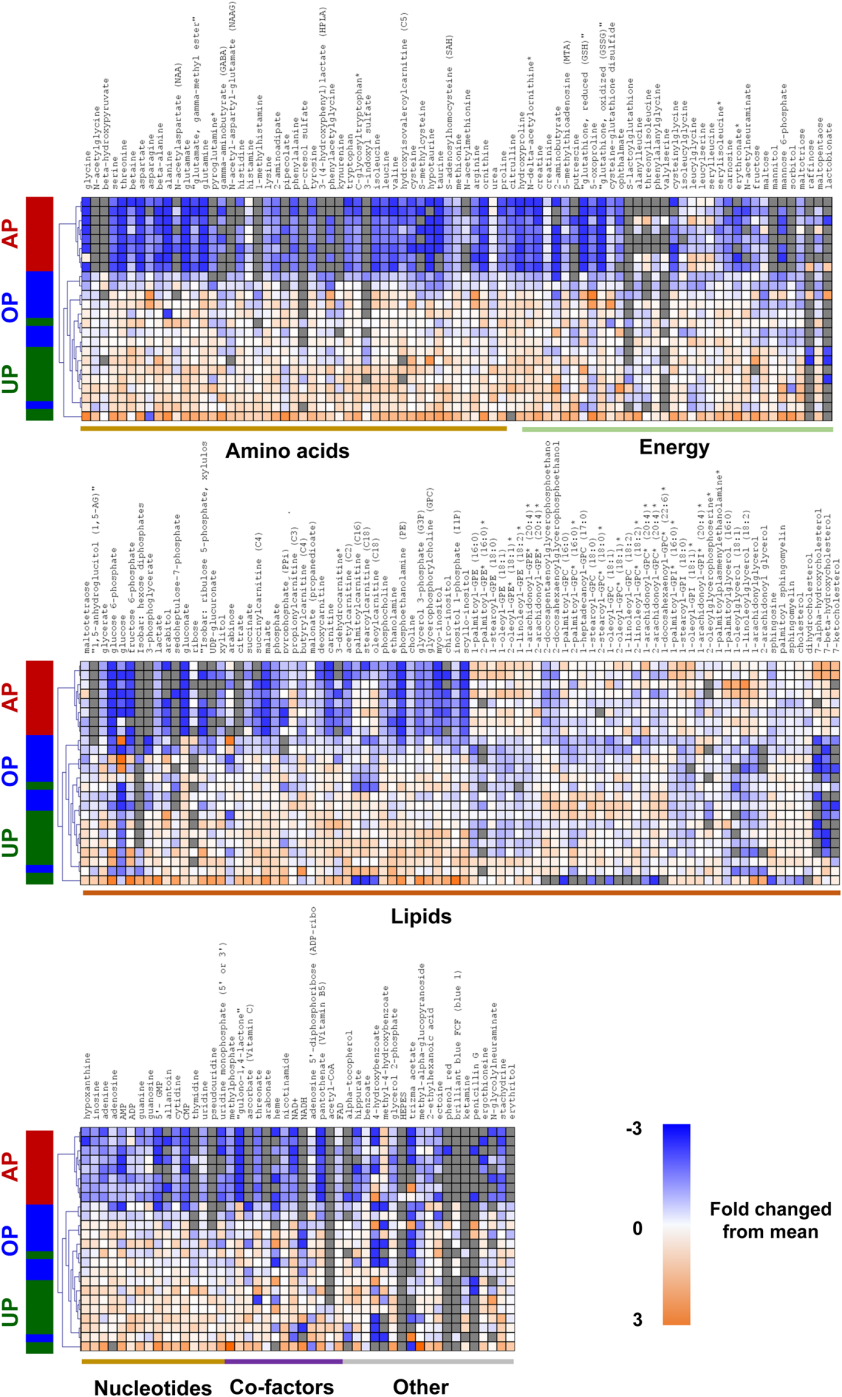
The metabolome of porcine saphenous vein. Hierarchical clustering analysis of porcine saphenous vein metabolites identified in unprepared (UP) vein, veins after standard preparation (AP) or optimized preparation (OP). Heatmap colors shaded blue indicate an increase relative to mean metabolite values while orange shades indicate a decrease relative to mean metabolite levels. Values shaded in grey were not detected. N = 8 per group.

Tissue level of 249 metabolites were detected in the PSV samples. The PCA scores plot revealed a clear separation between AP-PSV and UP-PSV (Fig. 3e). Unsupervised hierarchical clustering showed AP-PSV exhibiting similar metabolic perturbation detected in the HSV profiling (Fig. 5, Table 1). Significant changes were detected in 178 metabolites in AP-PSV compared to UP-PSV.

### Perturbation in redox homeostasis during vein graft preparation

**Figure 6:**
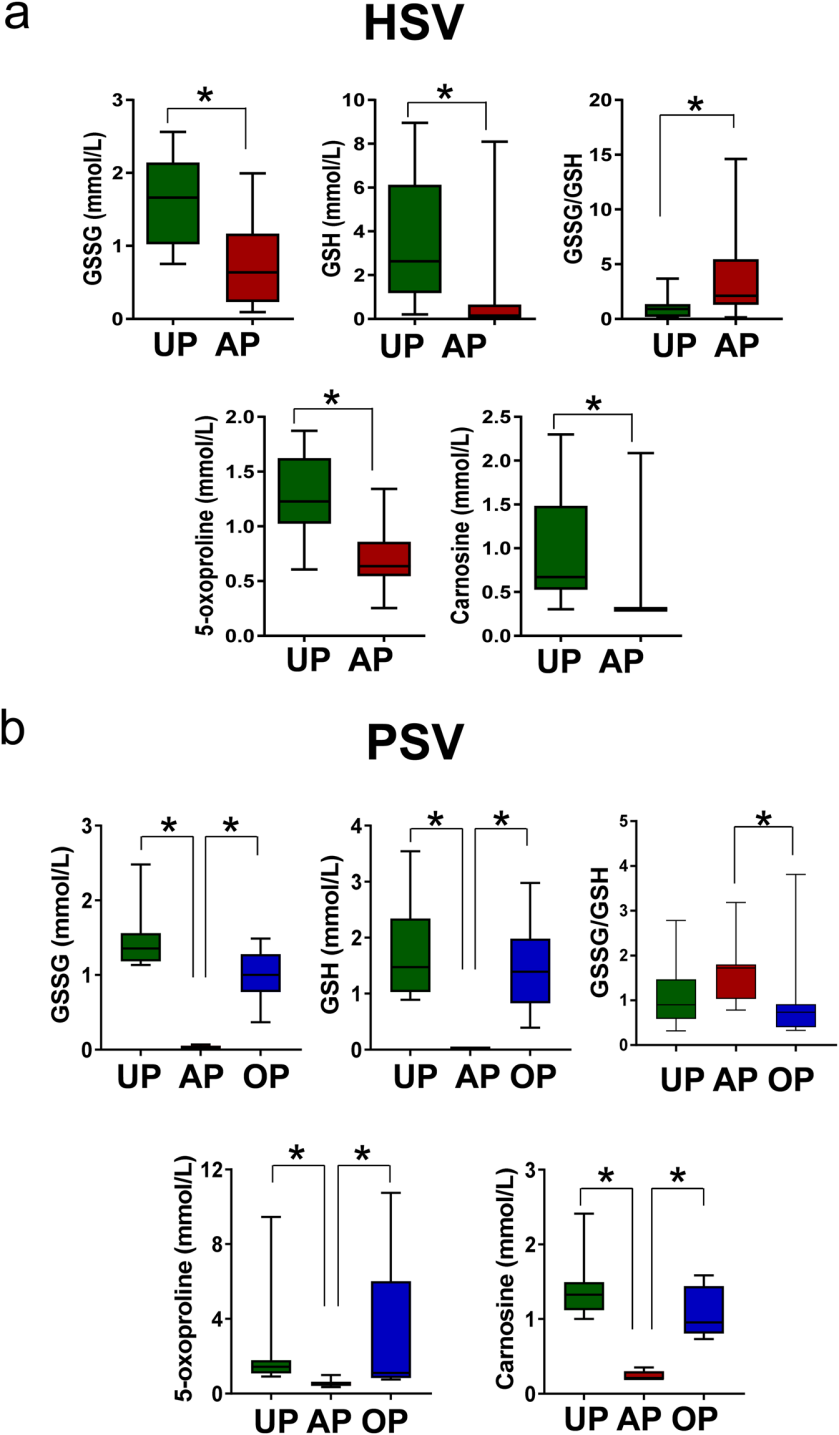
Redox metabolites in human and porcine saphenous vein before and after vein graft preparation. Levels of oxidized glutathione (GSSG), reduced glutathione (GSH), GSSG/GSH ratio, glutathione metabolite 5-oxoproline, and the anti-glycating agent carnosine in (**a**) HSV from CABG patients (n = 13) and (**b**) the PSV model (n = 8) unprepared (UP) or after preparations (AP in HSV; AP and OP in PSV). Values are expressed as box-and-whisker plots, *p ≤ 0.05.

Standard ‘back-table’ preparation was associated with increased oxidative stress observed in HSV^15^. Glutathione (GSH) is an important mediator of cellular redox homeostasis and plays crucial role in many cellular processes^27^. Metabolomics profiling revealed reduced levels of intermediates of GSH synthesis in AP-HSV (Fig. 6a) and AP-PSV (Fig. 6b). The ratio of oxidized-to-reduced glutathione (GSSG/GSH), an index of oxidative stress^27,28^, was increased in AP-HSV (Fig. 6a) and -PSV (Fig. 6b). Common anti-oxidant metabolites, 5-oxoproline and carnosine, were also reduced in AP-HSV and -PSV (Fig. 6).

### Oxidative injury and lipid peroxide formation with standard vein graft preparation

**Figure 7:**
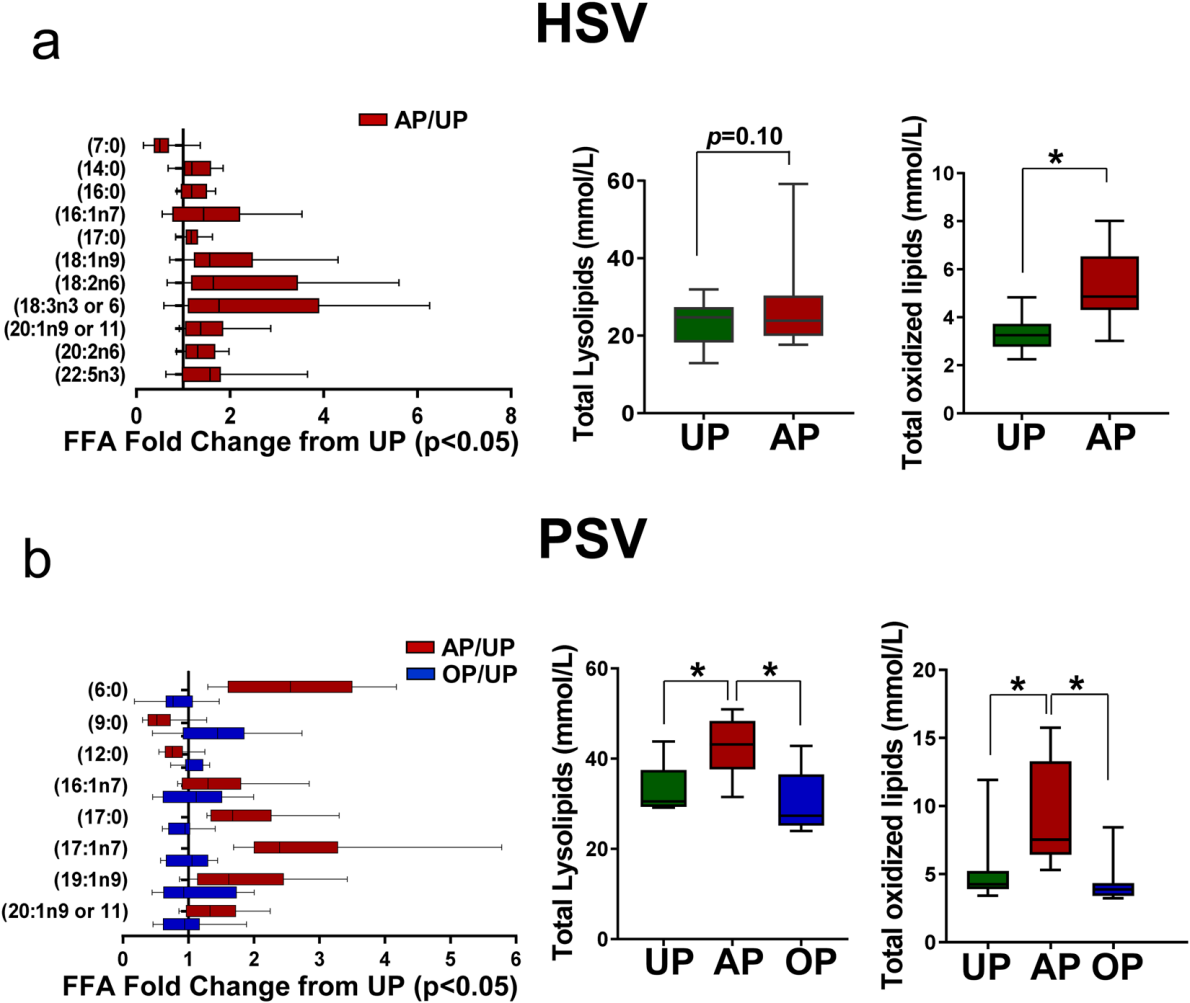
Lipid metabolites in human and porcine saphenous vein before and after vein graft preparation. Relative levels of free fatty acids that were significantly altered [HSV (AP/UP); PSV (AP/UP and OP/UP); *p* ≤ 0.05], total lysolipids, and total oxidized lipids in (**a**) HSV from CABG patients (n = 13) and (**b**) the PSV model (n = 8). Values are expressed as box-and-whisker plots, *p ≤ 0.05.

**Figure 8:**
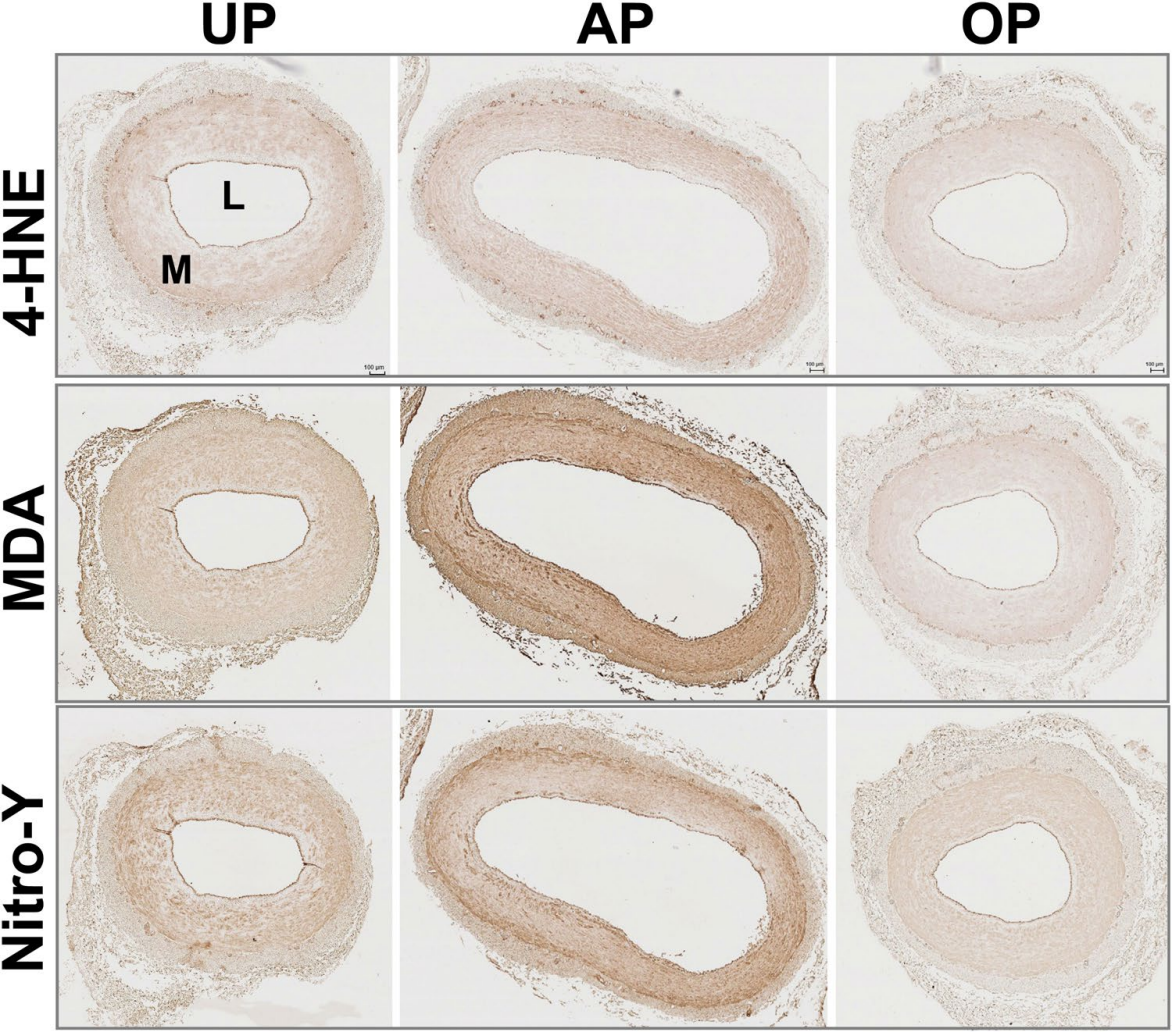
Levels of ROS-induced adducts in the porcine saphenous vein before and after vein graft preparation. PSV (n = 4) were harvested and prepared using the AP and OP technqiues, formalin-fixed and paraffin-embedded. Tissue sections were stained with antibodies to 4-hydroxynonenal (4-HNE, top row), malondialdehyde (MDA, middle row), or nitrotyrosine (Nitro-Y, bottom row). Whole slide imaging was performed at 20x. Scale bar = 100 µm. Brown = positive staining. Representative images of staining of tissues from 1 pig shown. UP, unprepared; AP, after standard vein graft preparation technique; OP, after optimized vein graft preparation technique; L, lumen; M, medial layer.

A considerable body of evidence implicates the production of reactive oxygen species (ROS) from increased oxidative stress and inflammation in the pathogenesis of vascular injury^29^. Metabolomics analysis revealed that standard vein graft preparation led to changes in phospholipid metabolism in both HSV and PSV (Figs 2 and 5). Metabolites detected in the lipid class including fatty acids (FA), lysolipids, eicosanoids and oxidized lipids showed an overall increase, indicative of phospholipid hydrolysis and oxidation. Of the FAs detected, 11 of 44 and 8 of 51 were increased in AP-HSV (Fig. 7a) and AP-PSV (Fig. 7b) tissues, respectively. Increase in total membrane lysolipids level in the AP tissues was approaching significance in AP-HSV (25 metabolites, p = 0.1; Fig. 7a) and significantly augmented in AP-PSV (28 metabolites, Fig. 7b). Oxidized phospholipids were elevated in the AP-HSV and -PSV (Fig. 7). Of particular note was the enhanced production of pro-inflammatory arachidonic and linoleic acid metabolites Consistent with increased levels of all FFAs in the AP AP-HSV and -PSV, levels of arachidonoyl (20:4)- and linoleoyl (18:2)-glycerophospholipid species were elevated (see Supplementary Fig. S1). In the vasculature, ROS produces a number of highly reactive bifunctional electrophiles derived from lipid peroxidation, including aldenals, malondialdehyde (MDA) and iso-levuglandins and levuglandins that alter vascular function that contributes to initiation and progression of vascular diseases such as atherosclerosis and hypertension^30,31^. Various derivatives of lipid peroxidation were found to be elevated in the AP tissues (see Supplementary Fig. S2). 12-hydroxyeicosatetraenoic acid (12-HETE) and13-hydroxyoctadecadienoic acid/9- hydroxyoctadecadienoic acid (13-HODE/9-HODE) were detected in AP-HSV (see Supplementary Fig. S2a). In the case of AP-PSV, hydroxycholesterols, hydroxyaldenal 4-hydroxynonenal (4-HNE) were elevated and augmented levels of 6-keto prostaglandin F1α (6-keto PGF1α) were present in 6 of the 8 animals, although it did not reach statistical significance (p = 0.14; see Supplementary Fig. S2). Oxidative stress-induced adduct formation of protein in the vein was visualized using immunohistochemistry. Nitrotyrosine (protein oxidation), 4-HNE and MDA (lipid peroxidation) adducts levels were markedly elevated in AP-PSV relative to UP-PSV (Fig. 8 and Supplemental Figs 6–8).

### Energy depletion resulting from standard vein graft preparation

**Figure 9:**
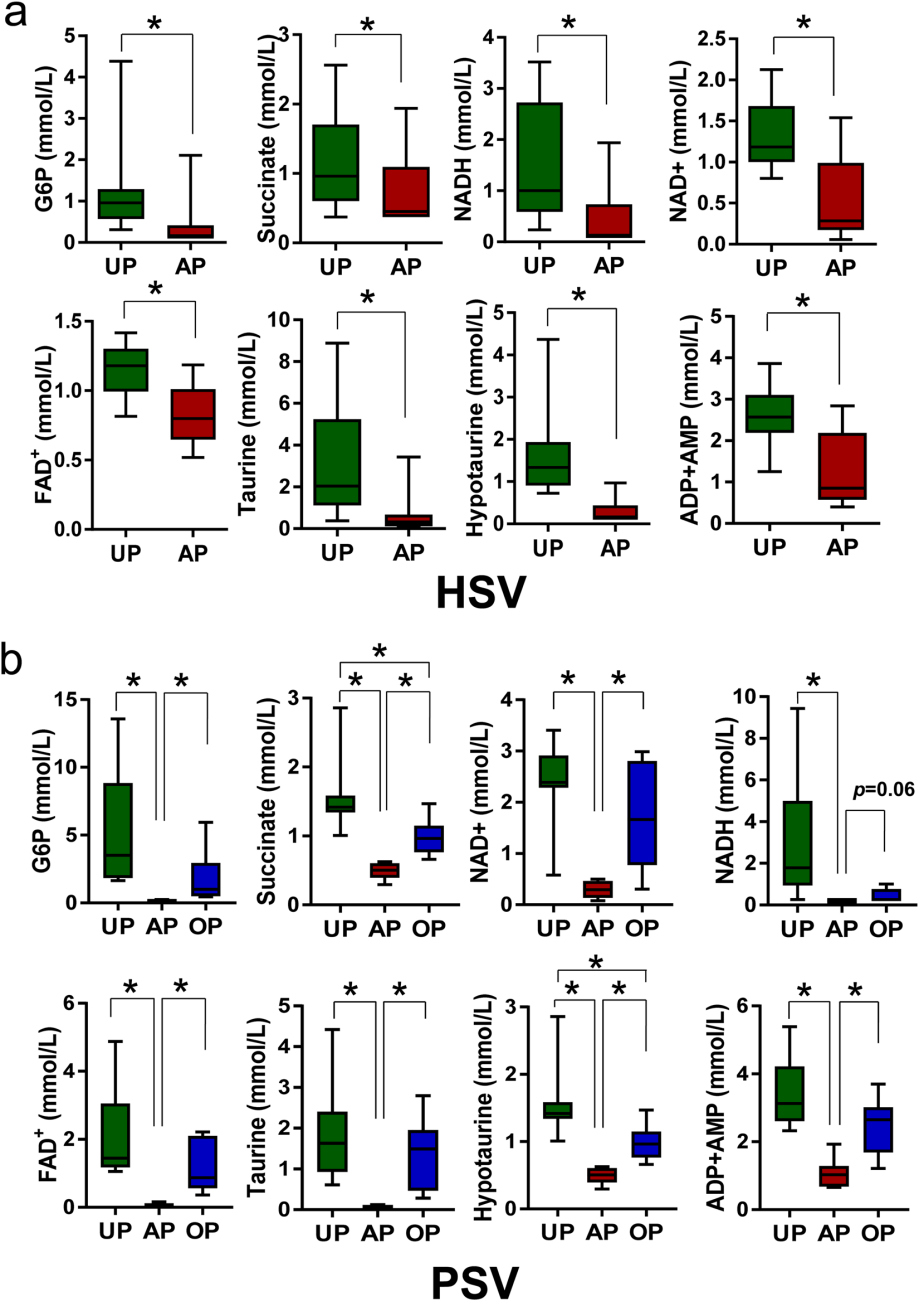
Energy metabolites in human and porcine saphenous vein before and after vein graft preparation. Levels of selected metabolites co-factors and intermediates central to the glycolysis and the Kreb’s cycle, and adenonucleotides in (**a**) HSV from CABG patients (n = 13) and (**b**) the PSV model (n = 8). Values are expressed as box-and-whisker plots, *p ≤ 0.05.

Metabolic alterations in glycolysis and TCA cycles in the AP tissues (Figs 2 and 5; see Supplementary Tables S1 and S2) suggested that there is a central energy depletion mediated by standard vein graft preparation. While glucose levels did not differ between UP- and AP-tissues from human or pigs, levels of several glycolytic intermediates (e.g. glucose-6-phosphate; Fig. 9) and TCA cycles (e.g. succinate; Fig. 9) were less abundant. There was also a decrease in the level of cofactors NAD^+^, NADH and FAD^+^ in the AP-HSV and -PSV (Fig. 9), which is critical in maintaining energy production. Levels of taurine, an antioxidant amino acid, and its derivative hypotaurine that can serve as cell energy source were almost abolished after standard vein graft preparation of both HSV and PSV (Fig. 9). These reductions in metabolites that are associated with energy metabolism contribute at least in part to decreased adenylate nucleotides level (ADP and AMP) in the AP tissues (Fig. 9); ATP was not detected in any of the tissues possibly due to the lack of production and rapid degradation into ADP and AMP. Alternatively, ATP may be released either through leaky disrupted membrane or receptor-mediated processes^32–34^.

### Impaired vasomotor function and metabolite abnormalities is prevented by the OP vein graft preparation routine

The OP graft preparation technique preserved vasocontractility (Fig. 3a and b), endothelial function (Fig. 3c) and vasorelaxation to SNP (Fig. 3d) in PSV. No statistically significant differences were observed upon comparison to UP-PSV. Vein morphology and endothelium also were preserved in the OP-PSV (Fig. 5). Thus, it is conceivable that mitigation of deleterious effects during graft preparation may correlate with normalization of metabolic alteration resulting from the AP preparation technique. Indeed, metabolite profiles of PSV prepared with the OP graft preparation closely resembled those of the UP-PSV (Figs 3e and 5). The PCA scores plot revealed a clear separation between AP-PSV and OP-PSV and an overlap of UP- and OP-PSV (Fig. 3e). Of the 178 metabolites that were different between UP- and AP-PSV, the OP routine preserved levels of 159 metabolites (AP vs. OP, p ≤ 0.05), as suggested by the overlap between UP- and OP-PSV tissues showed in PCA plot (Fig. 3e).

Redox homeostasis was preserved in OP-PSV. Levels of antioxidants did not diminish and the GSSG/GSH ratio was partially restored, similar to UP-PSV (Fig. 6b). Membrane disruption was also limited in OP-PSV. Levels of lysolipids and oxidized lipids were similar to UP suggesting that the OP vein graft preparation prevented the hydrolysis of phospholipids (Fig. 7b and see Supplementary Fig. S1), lipid peroxidation, and the conversion into 6-keto PGF1α in the PSV (see Supplementary Fig. S2). Relative levels of 4-HNE, MDA, and nitrotyrosine in OP-PSV were also similar to those of UP-PSV (Fig. 8 and Supplemental Figs S6–S8). In addition, disturbances the central energy metabolism were partially preserved in the OP-PSV (Fig. 9b).

## Discussion

The present study describes metabolic alterations that occur during standard preparation of human saphenous vein. An integrated picture of the complex and interdependent metabolic changes that occur in the conduits after manipulation in HSV was constructed. Decreased vasomotor function after standard back table preparation was associated with significant alterations in multiple metabolic pathways, most notably those reflective of oxidative stress, phospholipid hydrolysis, and energy depletion in HSV. Furthermore, a porcine model of controlled vein graft preparation was used to validate metabolic alteration with HSV and showed that an optimized preparation (OP) technique preserved graft function and prevented metabolic decompensation. These findings implicate an underappreciated role for metabolic injury in altered function of vascular conduits after standard back table preparation and justify simple, yet critically important changes to the methods commonly used to harvest and prepare conduits for CABG.

Vascular redox state has long been associated with vascular injury^29,35^. ROS generation in the vasculature occurs through multiple mechanisms with NADPH oxidase as the major source^36^. Increased oxidative stress mediates apoptosis, and influences of vascular remodeling and inflammatory responses^37,38^. In both human and the porcine models, 20- to 80-fold decreases in glutathione levels were observed with concomitant increase in GSSH/GSH levels, indicative of increased oxidative stress associated with the AP preparation, which includes high intraluminal distension pressures^1^. These findings are similar to those in a balloon-catheter distension model of carotid artery injury where glutathione levels dropped by 63% within 30 minutes after injury^39^; NADPH oxidase-mediated release of ROS has been described^40^. Mechanical stretch-induced ROS production has been linked to increased metalloprotease activity in VSMC which may contribute to increased extracellular matrix seen in intimal hyperplasia^41^.

The role of oxidized lipids in human diseases has been studied extensively^42^. Increase in oxidized lipids after standard graft preparation, as illustrated in both the human and porcine models (Fig. 7), is particularly noteworthy given their role in mediating acute inflammation and in the development of intimal hyperplasia and chronic atherosclerosis^43^. In the current study, significant alterations in oxidative stress and many of the pathways that interact with it were identified. Oxidation of fatty acids linked to glycerophospholipids leads to many different reaction products, depending on chain length and degree of unsaturation. Both hydroxy acids and their precursors, hydroxyperoxy acids, play an important role in maintenance of vascular homeostasis. *In vivo* (phospho)lipid oxidation occurs both enzymatically (catalyzed by lipoxygenases)^44,45^, and non-enzymatically (induced interaction with endogenously produced ROS by myeloperoxidases and NADPH oxidases)^46^. Arachidonic acid is released from membrane phospholipids in the course of inflammatory activation and converted into the pro-inflammatory eicosanoids which can be metabolized to prostaglandins and leukotrienes^30^. Arachidonyl-glycerphosphates (see Supplementary Fig. S1) and hydroxyperoxide fatty acid derivatives of arachidonic acid, 12-HETE (see Supplementary Fig. S2) were elevated in AP-HSV. Furthermore, arachidonic metabolites stimulate SMC migration^47^, a hallmark event that leads to intimal hyperplasia. Inhibition of 12-HETE production prevented IH in a rabbit graft model^48^.

The increase in lineoic acid was also noteworthy (see Supplementary Fig. S1). Oxidized fatty acids 9-hydroxyoctadecadienoic acid (9-HODE) and 13- hydroxyoctadecadienoic acid (13-HODE), elevated in AP-HSV (see Supplementary Fig. 2a), are enzymatic (15-lipoxygenase) and non-enzymatic end-products of linoleic acid oxidation, respectively. Endothelial cells produce and are the target of 13-HODE^49–51^, which regulates expression of inflammatory cellular adhesion molecules^52,53^. Oxidized products of membrane phospholipids can undergo further metabolism. The most abundant lipid peroxidation end products, 4-HNE and MDA, could interfere with cell function by forming protein or DNA adducts and exerting pro-inflammatory and pro-apoptotic pathway responses^54,55^. In AP-PSV tissue, the highly reactive 4-HNE were elevated (see Supplementary Fig. S2). Exposure to 4-HNE leads to depletion of GSH and endothelial activation^56^. Tissue staining showed increased 4-HNE, MDA levels in AP-PSV (Fig. 8 and Supplementary Fig. S3). Another common oxidative stress biomarker, nitrotyrosine, is also increased in AP-PSV (Fig. 8). Nitration of proteins in the vessel wall have been demonstrated and play a role in the cardiovascular pathologies^57^, one such example include attenuation of cGMP-dependent protein kinase activity which is critical for nitric oxide signaling^57,58^. Moreover, levels of oxysterols were increased in AP-PSV (see Supplementary Fig. S2), which may stimulate production of cytokines with proinflammatory and profibrogenic effects in the vessels^30^.

Cellular metabolic dysfunction and energy depletion exacerbate the response to injury. For example, the magnitude of perturbation of energy and oxidative stress metabolism were related to the severity of lung injuries in different animal models of acute respiratory distress syndrome^59^. In the scenarios of vascular injury described in this study, the metabolic changes found in AP-HSV and -PSV were characterized by dramatic shifts and deficits in nucleotides, amino acids and other energy-related metabolites (Figs 2 and 5). The decreased in energy state was evidenced by reduced glycolytic and TCA cycle activities that culminates in ATP depletion (Fig. 7). Additionally, taurine and hypotaurine, endogenous amino acids that serves as energy source and exert diverse array of biologic effects^60–63^, was depleted by 4- to 23-fold after standard preparation in both AP-HSV and -PSV (Fig. 9). Vascular effects of taurine based on experimental studies include anti-oxidation, anti-inflammatory, anti-apoptotic, and promotion of both smooth muscle- and endothelial-dependent vasoreactivity^62^.

**Figure 10:**
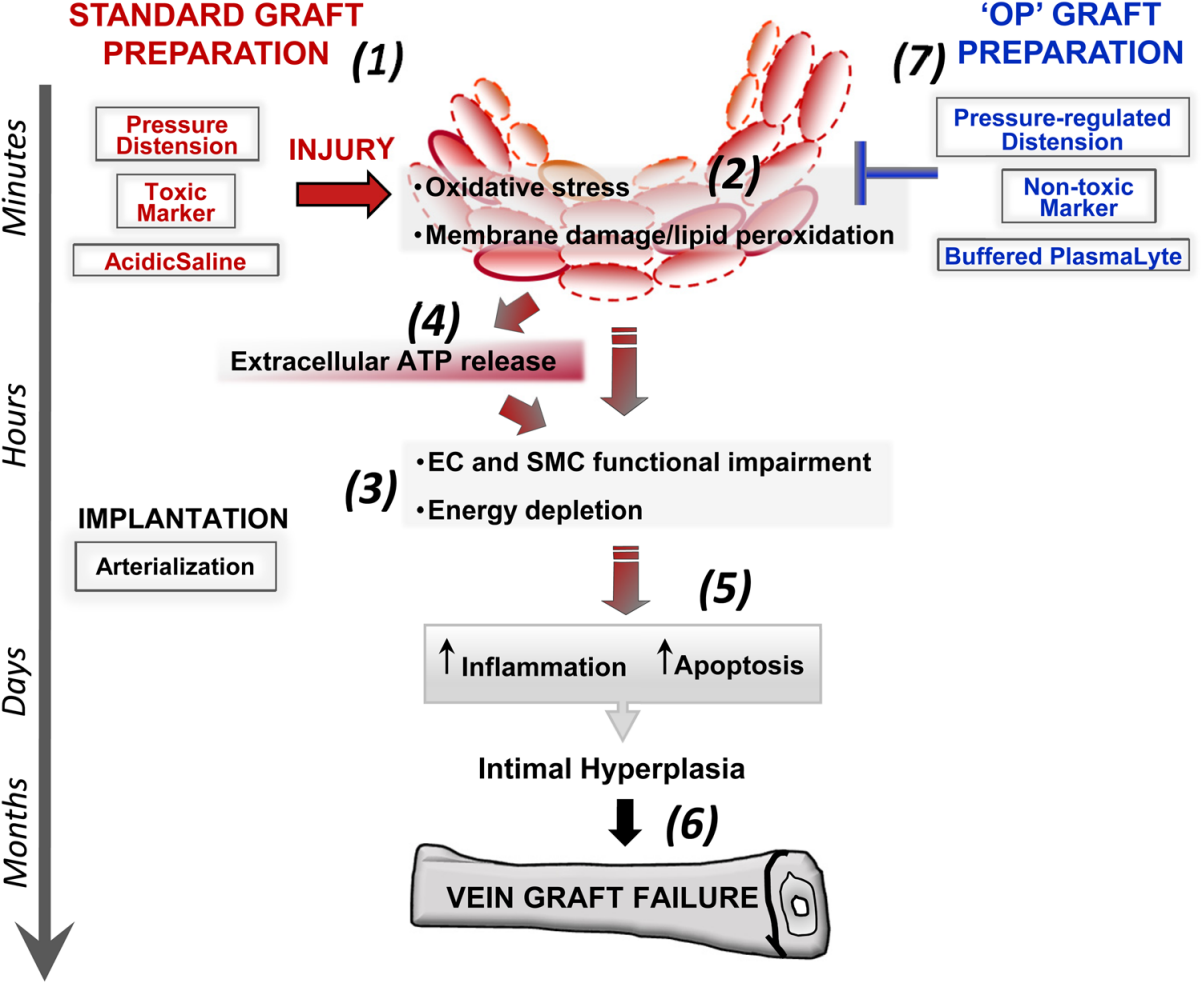
Model of saphenous vein injury with standard graft preparation and hypothesized routes of prevention. With standard saphenous vein graft preparation (SP) for CABG, pressure distension, acidic saline and off-label use of a surgical skin marker all contribute to vein graft injury (1, 2). Functional impairment of endothelial and smooth muscle cells and energy depletion (3), driven by oxidative stress, membrane damage/lipid peroxidation (2), results in extracellular ATP release (4) that exacerbate inflammation and apoptosis (5), ultimately leading to intimal hyperplasia and contributing to vein graft failure (6). With optimized graft preparation (OP) consisting of controlled pressure distension, the use of a non-toxic marker and buffered PlasmLyte solution (7), metabolic dysfunction is reduced, extracellular ATP release is minimized and vein graft injury is reduced.

In summary, metabolic profile analyses presented in this report suggest that standard vein graft preparation severely damages HSV (Fig. 10). Mechanical injury to the vein by subfailure longitudinal stretch during harvest^64^ and radial stretch resulting from pressure distension^1^ may lead to membrane injury. Chemical injury from off-label use of isopropyl-alcohol (50%) containing surgical skin marker also disrupts physical structure of cell membrane and has the potential to alter membrane protein activity^65^. Leakage of ATP and other soluble metabolites through disrupted cell membrane may contribute to the metabolic injury response. The release of ATP from damaged cells may also potentiate the injury by activating the ATP-sensitive, pore-forming purinergic P2X7 R^66,67^. Storage of the AP veins in unbuffered, acidic normal saline (pH 5.5–6.2) also contributes to metabolic dysfunction and decreased cellular viability^16^. Extracellular acidosis increases susceptibility to oxidant-induced apoptosis in vascular smooth muscle cells and has been reported to play a role in the pathophysiology of neuronal injury after stroke, cancer, and vascular disorders such as atherosclerosis and pulmonary hypertension^68–71^. Increased apoptosis has been observed in AP-HSV (lab unpublished results) and AP-PSV^17^. Thus, graft injury during preparation involves dynamic cascades of cellular response that reflect an intricate interdependence among acidosis, ROS imbalance, and membrane damage.

The metabolic deficits observed in the AP veins in the PSV model corroborated findings of the HSV. We demonstrated that these deleterious changes were mitigated by simple and straightforward technical approaches (OP). Induction in oxidative stress was prevented as the glutathione homeostasis were maintained (Fig. 6b) and markers of membrane disruption were minimized (Fig. 7b) in OP-PSV. Furthermore, central energy imbalance was abrogated when PSV were prepared using the OP technique (Fig. 9b). The OP technique utilizes a pressure-release valve^1^; brilliant blue FCF, a non-toxic water soluble P2X7R antagonist^22^, for graft marking; and storage in a balanced buffered electrolyte solution. While antioxidant such as ascorbic acid and resveratrol is under clinical evaluation as additive during vein graft storage^10,72^, vein graft preparation using straightforward technical improvements (OP technique) led to vasomotor function and metabolic parameters that were similar to unprepared tissues, suggesting that it is unlikely that further addition of anti-oxidant or anti-inflammatory drugs would demonstrate further improvement. The OP approach not only prevents physical (overstretch and overdistension) and chemical (isopropanol and acidosis)-induced membrane disruption, but conceivably limiting ATP loss and further cellular injury via P2X7R signaling (Fig. 10)^22^. FCF and other P2X7R inhibitors restore vasomotor function^5,22,64^ and prevent intimal hyperplasia in a rabbit interposition graft model suggesting that inhibition of P2X7R may have salutary effects in limiting propagation of vein graft injury^73^.

There are several limitations to this study. While porcine saphenous vein showed clear and beneficial improvements in vasomotor function and metabolic parameters with OP technique, it is unclear if similar trends will occur when OP is applied to HSV. Current protocols do not allow for procurement of adequate UP-HSV to apply the OP technique. Existing metabolic data are limited to qualitative analysis, which must be validated against quantitative measures in future. While several putative molecular targets were identified by this layered analysis, specific target validation will be relegated to future studies. Normalization of vasomotor function and metabolic phenotypes occurred with OP; however, long term and clinical effects (intimal hyperplasia, vein graft failure) of reducing the acute injury and injury response requires further study.

## Conclusions

The association of vasomotor dysfunction, increased oxidative stress, inflammation, and intimal hyperplasia with current vein preparation techniques have been described by our laboratory and others for decades; yet understanding of the molecular basis for the response to injury and the adoption of techniques to limit injury have been limited. This study correlates global metabolic derangement with preparation injury and demonstrates that the metabolic biosignature of saphenous vein can be preserved during vein graft preparation using simple, straightforward technical improvements (OP technique). Thus, strategies aimed at reducing injury during graft harvest and preparation represents a straightforward and viable strategy to preserve conduit function and viability, and possibly improve graft patency. The metabolic profiles identified in this study suggest that increased oxidative stress, membrane injury, and loss of energy reserves are initial components of the early response to mechanical and chemical injury associated with surgical vein graft harvest and preparation. The saphenous vein represents an organ that is transplanted into key vascular beds to prevent limb loss, myocardial infarction, and death. Increasing our understanding of and limiting injury to the conduit, and to preserve viability is required prior to implementing further therapeutic interventions to preserve vein graft patency.

## Electronic supplementary material

Supplemental Information
