# Supplementary material for: Limiting Injury During Saphenous Vein Graft Preparation For Coronary Arterial Bypass Prevents Metabolic Decompensation
Source: Sci Rep. 2017 Oct 27;7:14179. doi: 10.1038/s41598-017-13819-w (PMC5660200; doi:10.1038/s41598-017-13819-w)
Supplement: Supplementary file 1 — Supplemental Information [file 41598_2017_13819_MOESM1_ESM.doc]

**SUPPLEMENTARY INFORMATION**

**Limiting Injury During Saphenous Vein Graft Preparation For Coronary Arterial Bypass Prevents Metabolic Decompensation.**

Joyce Cheung-Flynn^1^, Jun Song^1^, Igor Voskresensky^1^, Eric S. Wise^1^, Yapu Liu ^1, 2^, Yanhua Xiong^1^, Susan S. Eagle^3^, Colleen M. Brophy^1^, and C. Robb Flynn^1^,*

**Supplementary Methods**

The non-targeted metabolic profiling platform employed for this analysis combined three independent platforms: ultrahigh performance liquid chromatography/tandem mass spectrometry (UHPLC/MS/MS2) optimized for basic species, UHPLC/MS/MS2 optimized for acidic species, and gas chromatography/mass spectrometry (GC/ MS). Saphenous vein segments (50 mg – stored frozen in liquid nitrogen with endothelium intact) were processed essentially as described previously.^1,2^ For each sample, 100 µL of organic homogenate was used for analyses. Using an automated liquid handler (Hamilton LabStar, Salt Lake City, UT), protein was precipitated from the homogenate with methanol that contained four standards to report on extraction efficiency. The resulting supernatant was split into equal aliquots for analysis on the three platforms. Aliquots, dried under nitrogen and vacuum-desiccated, were subsequently either reconstituted in 50L 0.1% formic acid in water (acidic conditions) or in 50 µl 6.5mM ammonium bicarbonate in water, pH 8 (basic conditions) for the two UHPLC/ MS/MS2 analyses or derivatized to a final volume of 50 µl for GC/MS analysis using equal parts bistrimethyl-silyltrifluoroacetamide and solvent mixture acetonitrile:dich loromethane:cyclohexane (5:4:1) with 5% triethylamine at 60°C for one hour. In addition, three types of controls were analyzed in concert with the experimental samples: aliquots of a well-characterized human plasma pool served as technical replicates throughout the data set, extracted water samples served as process blanks, and a cocktail of standards spiked into every analyzed sample allowed instrument performance monitoring. Experimental samples and controls were randomized across platform run days.

For UHLC/MS/MS2 analysis, aliquots were separated using a Waters Acquity UPLC (Waters, Millford, MA) and analyzed using an LTQ mass spectrometer (Thermo Fisher Scientific, Inc., Waltham, MA) which consisted of an electrospray ionization (ESI) source and linear iontrap (LIT) mass analyzer. The MS instrument scanned 99–1000 m/z and alternated between MS and MS2 scans using dynamic exclusion with approximately 6 scans per second. Derivatized samples for GC/MS were separated on a 5% phenyldimethyl silicone column with helium as the carrier gas and a temperature ramp from 60°C to 340°C and then analyzed on a Thermo-Finnigan Trace DSQ MS (Thermo Fisher Scientific, Inc.) operated at unit mass resolving power with electron impact ionization and a 50–750 atomic mass unit scan range.

Metabolites were identified by automated comparison of the ion features in the experimental samples to a reference library of 369 chemical standard entries that included retention time, molecular weight (m/z), preferred adducts, and in-source fragments as well as associated MS spectra, and were curated by visual inspection for quality control using software developed at Metabolon.^3^

For statistical analyses and data display purposes, any missing values were assumed to be below the limits of detection and these values were imputed with the compound minimum (minimum value imputation). Statistical analysis of log-transformed data was performed using “R” (http://cran.r-project.org/). Welch's t-tests were performed to compare data between experimental groups. A p-value of < 0.05 was considered statistically significant and multiple comparisons were accounted for by estimating the false discovery rate (FDR) using q-values.^4^

**Identification and justification for excluding two HSV sample pairs**

Metabolites identified as outliers by ROUT (robust regression and outlier removal) analysis (Graphpad Prism 6.0) in subjects 1 and 7. Pre (A) and post (B) manipulation metabolite levels shaded in pink exhibited a Q value of 1% (outliers).


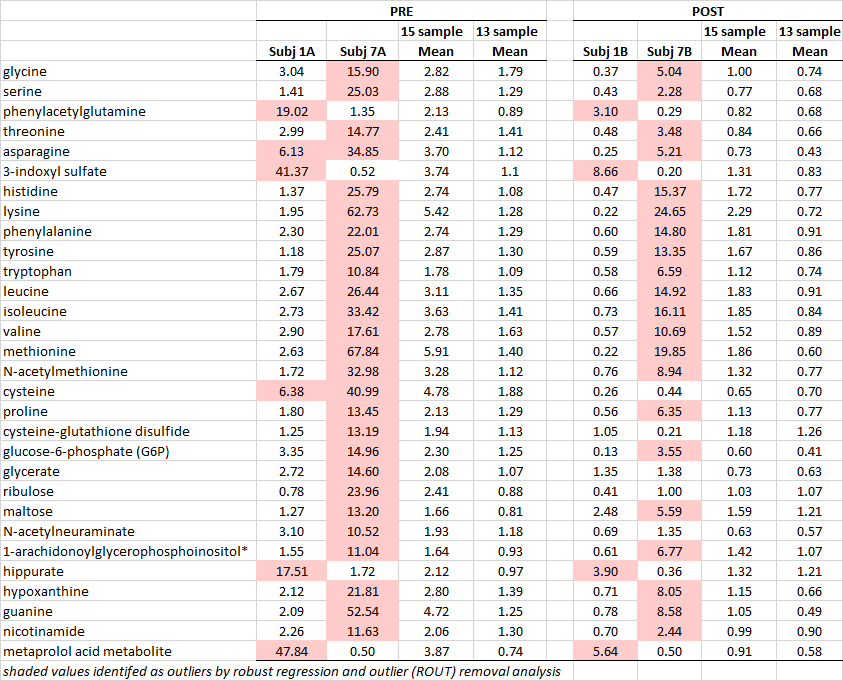


**Supplementary references**

1. Ohta T, Masutomi N, Tsutsui N, et al. Untargeted metabolomic profiling as an evaluative tool of fenofibrate-induced toxicology in Fischer 344 male rats. Toxicol Pathol 2009;37:521-35.

2. Evans AM, DeHaven CD, Barrett T, Mitchell M, Milgram E. Integrated, nontargeted ultrahigh performance liquid chromatography/electrospray ionization tandem mass spectrometry platform for the identification and relative quantification of the small-molecule complement of biological systems. Anal Chem 2009;81:6656-67.

3. Dehaven CD, Evans AM, Dai H, Lawton KA. Organization of GC/MS and LC/MS metabolomics data into chemical libraries. J Cheminform 2010;2:9. PMCID:PMC2984397

4. Storey JD, Tibshirani R. Statistical significance for genomewide studies. Proc Natl Acad Sci U S A 2003;100:9440-5. PMCID:PMC170937


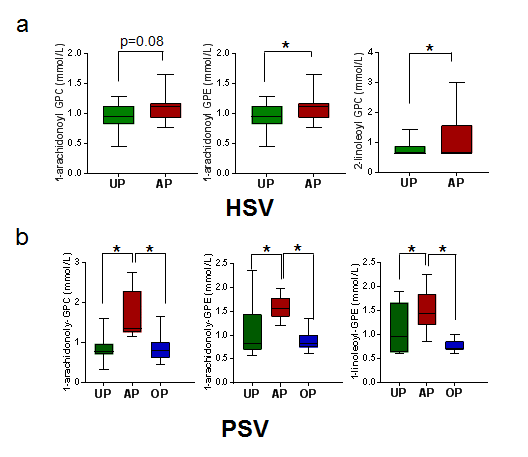


**Supplementary Figure S1. Levels of oxidative stress lysolipid markers in human and porcine saphenous vein before and after vein graft preparation.** Levels of significantly altered arachidonyl- and linoleoyl-glycerophosphates (GPC or GPE) in (A) HSV from CABG patients (n=13) and (B) the PSV model (n=8). Values are expressed as box-and-whisker plots, **p*≤0.05.


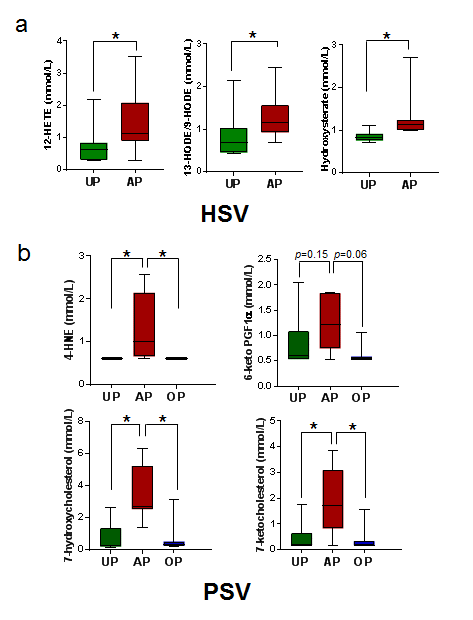


**Supplementary Figure S2. Levels of oxylipins in human and porcine saphenous vein before and after vein graft preparation.** (A) Levels of eicosanoids (12-HETE, 13-HODE/9-HODE) and hydroxysterate in HSV from CABG patients (n=13). (B) Levels of lipid peroxidation products 4-HNE and 6-keto PGF1α, and oxysterols in the PSV model (n=8). Values are expressed as box-and-whisker plots, **p*≤0.05.

**
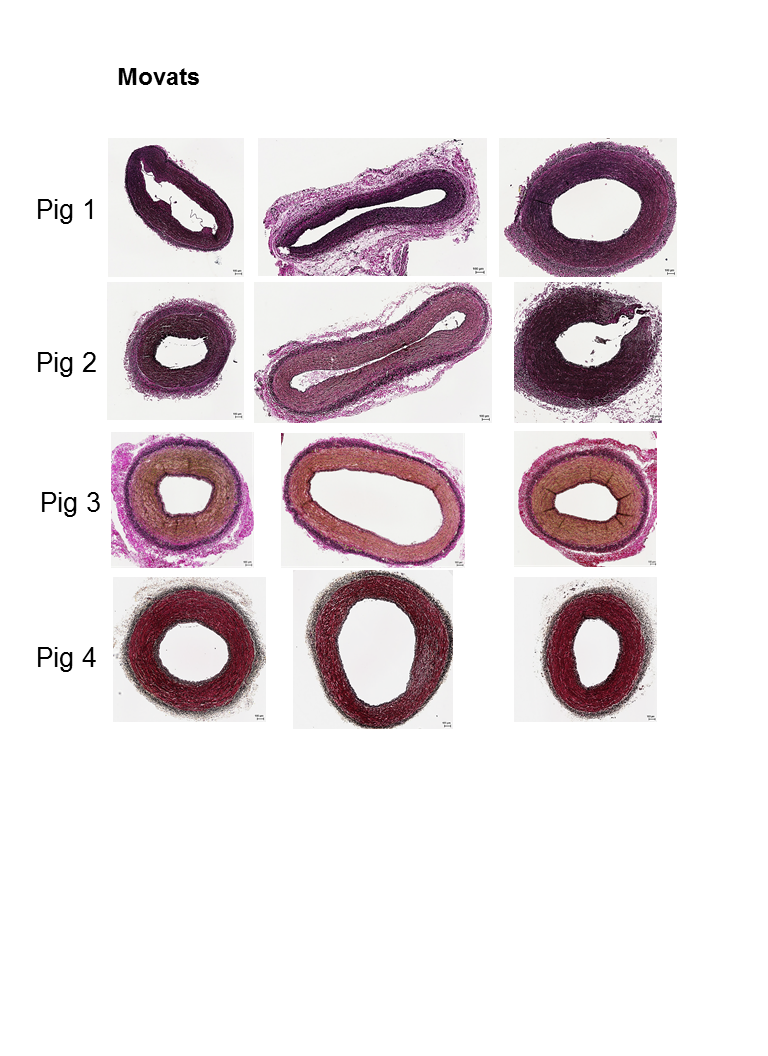
**

**Supplemental Figure S3. Histomorphology of the porcine saphenous vein before and after vein graft preparation in the PSV model.** PSV (n=4) were harvested and prepared using the AP and OP technqiues, formalin-fixed and paraffin-embedded. Tissue sections were stained to visualize histomorphology with Van Gieson’s (Pigs 1-3) or Movat’s stain. Whole slide imaging was performed at 20x. Scale bar=100
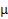
m. UP, unprepared; AP, after standard vein graft preparation technique; OP, after optimized vein graft preparation technique, L, lumen; M, medial layer.

**
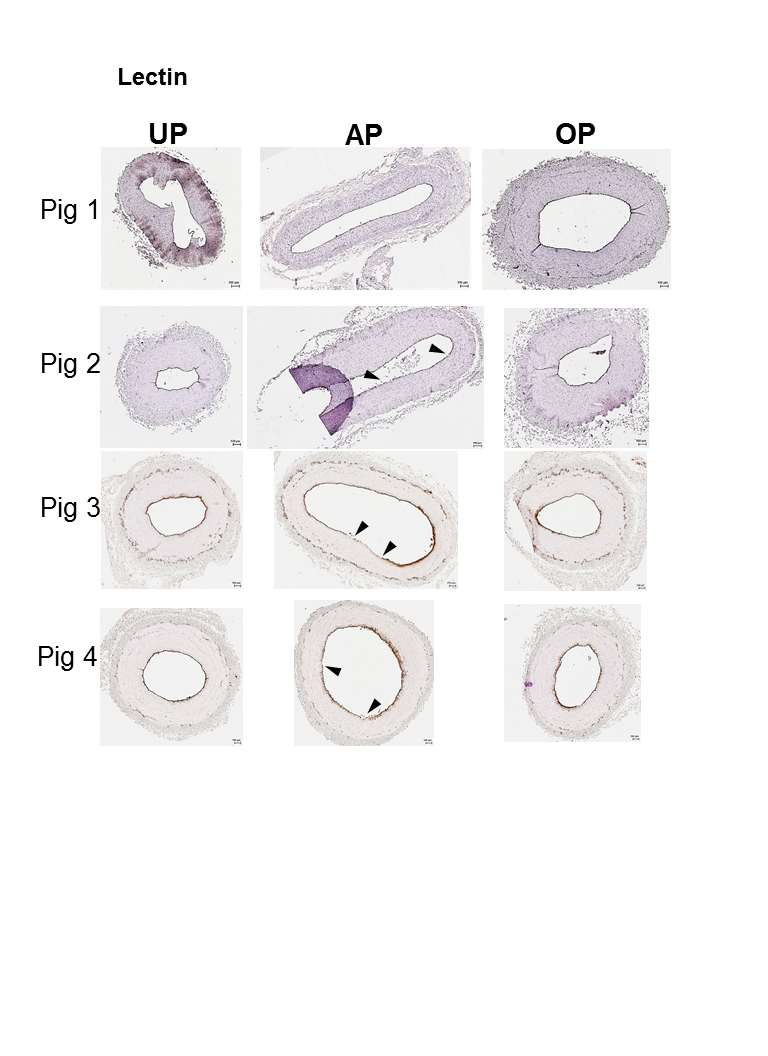
**

**Supplemental Figure S4. Immunohistomorphology of the porcine saphenous vein before and after vein graft preparation in the PSV model.** PSV (n=4) were harvested and prepared using the AP and OP technqiues, formalin-fixed and paraffin-embedded. Tissue sections were stained with lectin (Dolichos biflorus agglutinin) to examine endothelial integrity. Whole slide imaging was performed at 20x. Scale bar=100
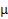
m. Brown=positive staining. UP, unprepared; AP, after standard vein graft preparation technique; OP, after optimized vein graft preparation technique, L, lumen; M, medial layer. Arrows indicate endothelial disruption.

**
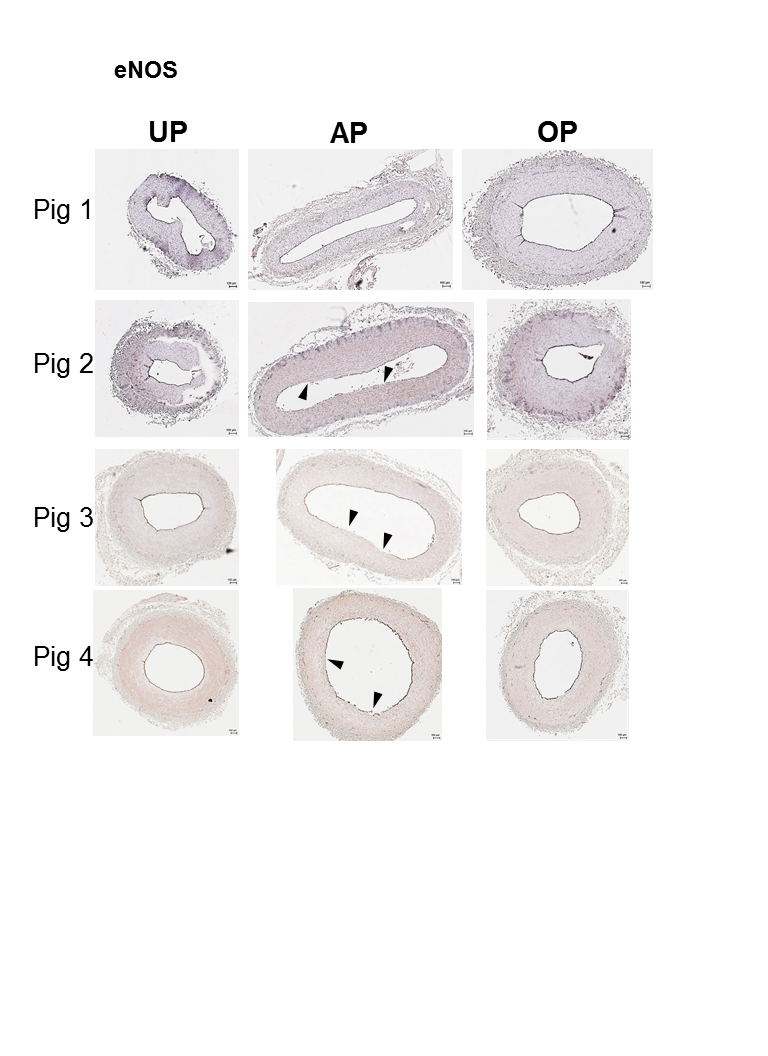
**

**Supplemental Figure S5. Immunohistomorphology of the porcine saphenous vein before and after vein graft preparation in the PSV model.** PSV (n=4) were harvested and prepared using the AP and OP technqiues, formalin-fixed and paraffin-embedded. Tissue sections were stained with antibody against endothelial nitric oxide synthase (eNOS) to examine endothelial integrity. Whole slide imaging was performed at 20x. Scale bar=100
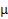
m. Brown=positive staining, blue-nuclei. UP, unprepared; AP, after standard vein graft preparation technique; OP, after optimized vein graft preparation technique, L, lumen; M, medial layer. Arrows indicate endothelial disruption.

**
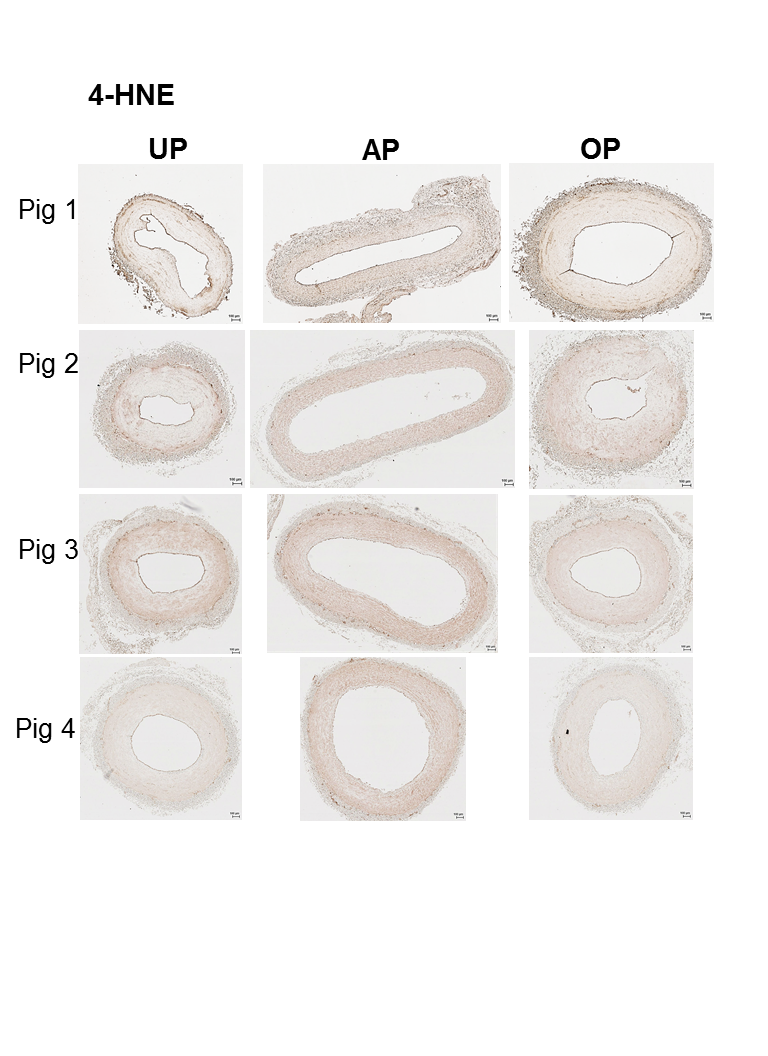
**

**Supplemental Figure S6. Immunohistomorphology of the porcine saphenous vein before and after vein graft preparation in the PSV model.** PSV (n=4) were harvested and prepared using the AP and OP technqiues, formalin-fixed and paraffin-embedded. Tissue sections were stained with antibody against 4-hydroxynonenal (4-HNE). Whole slide imaging was performed at 20x. Scale bar=100
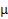
m. Brown=positive staining. UP, unprepared; AP, after standard vein graft preparation technique; OP, after optimized vein graft preparation technique, L, lumen; M, medial layer. Arrows indicate endothelial disruption observed in the AP tissues.

**
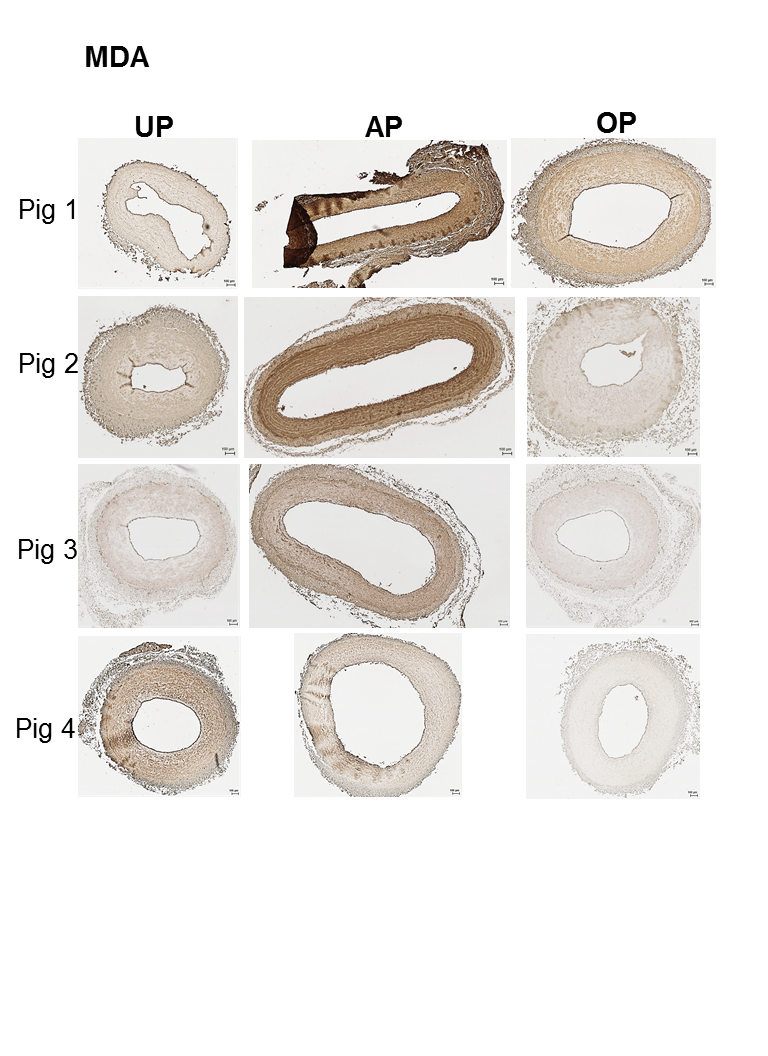
**

**Supplemental Figure S7. Immunohistomorphology of the porcine saphenous vein before and after vein graft preparation in the PSV model.** PSV (n=4) were harvested and prepared using the AP and OP technqiues, formalin-fixed and paraffin-embedded. Tissue sections were stained with antibody against malondialdehyde (MDA). Whole slide imaging was performed at 20x. Scale bar=100
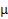
m. Brown=positive staining. UP, unprepared; AP, after standard vein graft preparation technique; OP, after optimized vein graft preparation technique, L, lumen; M, medial layer. Arrows indicate endothelial disruption observed in the AP tissues.

**
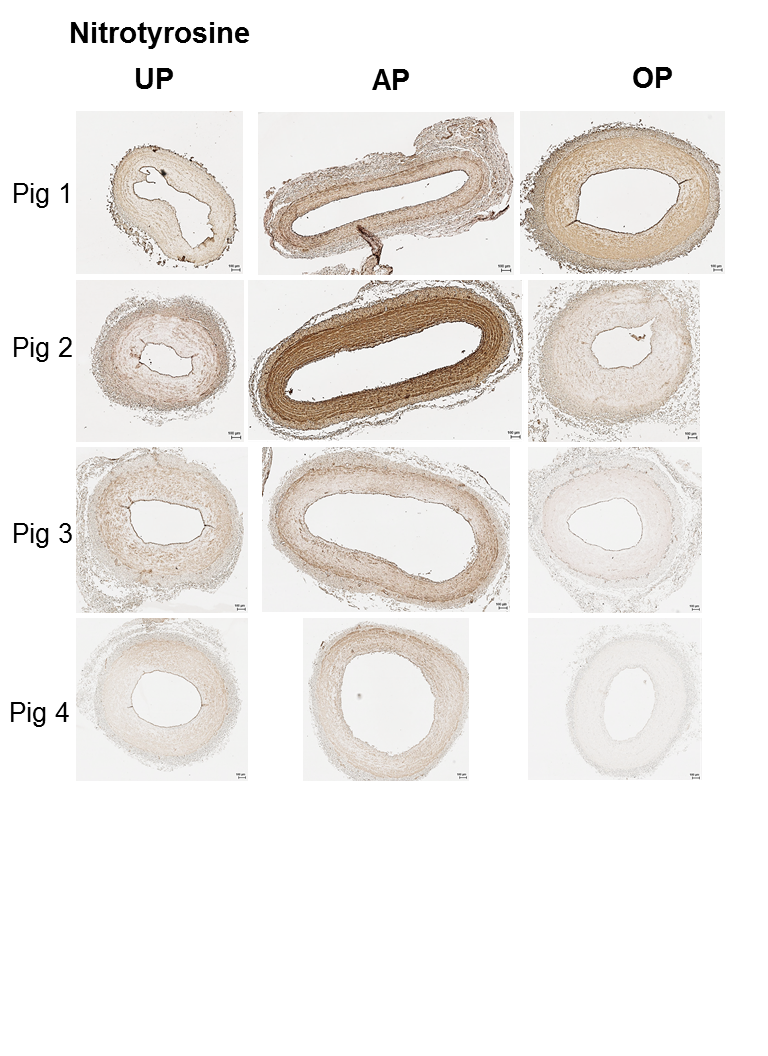
**

**Supplemental Figure S8. Immunohistomorphology of the porcine saphenous vein before and after vein graft preparation in the PSV model.** PSV (n=4) were harvested and prepared using the AP and OP technqiues, formalin-fixed and paraffin-embedded. Tissue sections were stained with antibody against nitrotyrosine (nitro-Y). Whole slide imaging was performed at 20x. Scale bar=100
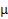
m. Brown=positive staining. UP, unprepared; AP, after standard vein graft preparation technique; OP, after optimized vein graft preparation technique, L, lumen; M, medial layer. Arrows indicate endothelial disruption observed in the AP tissues
